# An efficient method for mining cross-timepoint gene regulation sequential patterns from time course gene expression datasets

**DOI:** 10.1186/1471-2105-14-S12-S3

**Published:** 2013-09-24

**Authors:** Chun-Pei Cheng, Yu-Cheng Liu, Yi-Lin Tsai, Vincent S Tseng

**Affiliations:** 1 Department of Computer Science and Information Engineering, National Cheng Kung University, No.1, University Road, Tainan City 701, Taiwan; 2 Department of Environmental and Occupational Health, National Cheng Kung University, No.1, University Road, Tainan City 701, Taiwan; 3 Institute of Medical Informatics, National Cheng Kung University, No.1, University Road, Tainan City 701, Taiwan

## Abstract

**Background:**

Observation of gene expression changes implying gene regulations using a repetitive experiment in time course has become more and more important. However, there is no effective method which can handle such kind of data. For instance, in a clinical/biological progression like inflammatory response or cancer formation, a great number of differentially expressed genes at different time points could be identified through a large-scale microarray approach. For each repetitive experiment with different samples, converting the microarray datasets into transactional databases with significant singleton genes at each time point would allow sequential patterns implying gene regulations to be identified. Although traditional sequential pattern mining methods have been successfully proposed and widely used in different interesting topics, like mining customer purchasing sequences from a transactional database, to our knowledge, the methods are not suitable for such biological dataset because every transaction in the converted database may contain too many items/genes.

**Results:**

In this paper, we propose a new algorithm called *CTGR-Span *(Cross-Timepoint Gene Regulation Sequential pattern) to efficiently mine *CTGR-SPs *(Cross-Timepoint Gene Regulation Sequential Patterns) even on larger datasets where traditional algorithms are infeasible. The *CTGR-Span *includes several biologically designed parameters based on the characteristics of gene regulation. We perform an optimal parameter tuning process using a GO enrichment analysis to yield *CTGR-SPs *more meaningful biologically. The proposed method was evaluated with two publicly available human time course microarray datasets and it was shown that it outperformed the traditional methods in terms of execution efficiency. After evaluating with previous literature, the resulting patterns also strongly correlated with the experimental backgrounds of the datasets used in this study.

**Conclusions:**

We propose an efficient *CTGR-Span *to mine several biologically meaningful *CTGR-SPs*. We postulate that the biologist can benefit from our new algorithm since the patterns implying gene regulations could provide further insights into the mechanisms of novel gene regulations during a biological or clinical progression. The Java source code, program tutorial and other related materials used in this program are available at http://websystem.csie.ncku.edu.tw/CTGR-Span.rar.

## Background

Over the past decade, a great number of studies on time course issue have become increasingly important since most clinical/biological events, such as infection-related chronic/acute inflammatory responses [1-3], drug treatment-related experiments [4], cell cycle-arrest [5] or other important issues [6], require a period of time in which aberrant alterations in gene expression would lead to different outcomes. Therefore, through performing a consecutive monitoring of massive gene expressions and discovering their regulations during clinical/biological manifestations, the hidden layer of biological mechanisms could be unveiled. However, to our knowledge, these is no effective method can handle this issue although the high-throughput microarray is a powerful tool and has been widely utilized to efficiently detect differentially expressed genes among a group of patients in a time course experiment [3,4]. These authors only focused on how to identify differentially expressed genes varied with time but actually we did not know whether these genes are associated with each other or not. Their results did not show the valuable information.

Sequential pattern mining is one of the most important topics in the field of data mining, especially for the database systems. The fundamental meaning of a sequential pattern refers to a set of singleton frequent items/differentially expressed genes that are followed by another set of items/differentially expressed genes in the time-stamp ordered transaction. Therefore, once the potential gene regulations occurred in a period of time, it could be identified by mining such sequential patterns from a dataset-converted database. Referring to previous studies, several parental algorithms using different computational designs, such as *AprioriAll *[7], *SPADE *[8] and *PrefixSpan *[9], have been successfully proposed and used for different databases to discover their own sequential patterns. The *apriori*-like (level-wise) *GSP *[10] and pattern-growth-based *Prefix-growth *[11] as well as *DELISP *[12] are evolutionarily designed incorporating with many constraints such as the size of gap among the sequence-involved singleton items, or a time interval within which items are observed as belonging to the same transaction even if they originate from different transactions. Besides, any possible subpatterns derived from each parental sequential pattern also satisfy the user-set constraint values. This property is called *downward closure *[7-12]. Therefore, any possible subpatterns of each sequential pattern, particularly for the longer ones, need to be generated during the decomposing process that is time-consuming and space-exhausting. Once both shorter and longer sequential patterns have the same occurrence times across all transactions in the database, i.e., *closed sequential patterns*, the shorter ones will be eliminated from the final resulting patterns. For this purpose, some newer algorithms like incorporating with constraints, *CTSP *[13], and without constraints, *CloSpan *[14], were then designed to tackle this problem. In addition to these traditional algorithms, an increasing number of extended methods have also been performed on some interesting topics. For example, an algorithm called *WSpan *[15] could be used to determine *weighted sequential patterns *from a transactional database, and the *MAGIIC *[16] was designed to discover the structure motifs from protein sequences. However, to the best of our knowledge, all of the aforementioned methods are not suitable for the widely used microarray data, as a large-scale DNA microarray-based platform normally consists over tens of thousands of probes/genes, e.g., over 45,000 probes/genes in rice and over 20,000 probes/genes in human arrays. A set of differentially expressed genes (significant singleton gene items) on a single array could be individually considered as a single transaction. In that way, each transaction (each time point contained gene items) may contain too many significant singleton gene items after converting the numeric datasets into the format (discrete) of transactional databases [17]. This is called a long transaction issue. However, to date, there exists no method which can efficiently handle such kind of issue. Actually, a lot of items would frequently occur at most time points. They are similar to the housekeeping genes, which are very insensible to an extracellular stimulus; instead, they play critical roles as maintenance genes in the basic cellular functions [18]. Moreover, mining sequential patterns containing too many such items may increase the difficulty in interpreting the resulting gene regulations. The performance of the preceding sequential pattern mining methods would also be limited to these simultaneous items.

In this paper, we propose a new algorithm called *CTGR-Span *(Cross-Timepoint Gene Regulation Sequential pattern) with some biologically designed parameters to solve the issue mentioned above by mining *CTGR-SPs *(Cross-Timepoint Gene Regulation Sequential Patterns). The *CTGR-Span *ensures that all of the resulting patterns imply gene regulations, which take place across different time points during the course of biological observations. The method is an extended and improved version of our previous paper [19] presented in the *2012 IEEE International Conference on Bioinformatics and Biomedicine *(*BIBM*). The most important changes include: first, we designed a new optimal parameter tuning procedure for the proposed algorithm to ideally determine suitable conditions in pattern mining. The procedure has a merit that there is no need to additionally compute the standard deviation of time intervals in a time course dataset. Based on this design, then we compared our method with two representative sequential pattern mining algorithms, namely *GSP *and *PrefixSpan*, in execution efficiency and effectiveness. The resulting patterns were validated using a manual literature survey and an automatic Gene Ontology enrichment analysis [20]. Finally, more explanations for the proposed algorithm have also been added to this paper like i) providing complete examples for readily understanding both our proposed algorithm and the new parameter tuning procedure, and ii) performing more experimental results on the two publicly available human disease-related time course microarray datasets [3,4].

The rest of this paper is organized as follows. The proposed method and materials for analysis are described in Methods. In Results and Discussion, we give the experimental results of the proposed method on two time course gene expression datasets. Concluding remarks are given in Conclusions.

## Methods

In this section, we introduce how to efficiently discover *CTGR-SPs *(Cross-Timepoint Gene Regulation Sequential Patterns) from a time course microarray dataset through 3 main parts: i) an introduction to the experimental background of 2 input microarray datasets, ii) how to convert a numeric dataset into a transactional database, and iii) the kernel of the *CTGR-Span *(Cross-Timepoint Gene Regulation Sequential pattern) and its required biologically designed arguments.

### Input microarray datasets

We tested this paper presenting method using the same input datasets as our previous works [19]. In brief, 2 time course gene expression microarray datasets (GSE6377 [3] and GSE11342 [4]) were downloaded from the GEO database. In GSE6377, McDunn *et al*. attempted to detect 8,793 transcriptional changes in 11 ventilator-associated pneumonia patients' leukocytes across 10 time points. For the other GSE11342, Taylor *et al*. monitored 22,283 gene expression changes in peripheral blood monocytes of 20 hepatitis C virus infected patients across the first 10 weeks right after treating with the Peg-interferon alfa-2b plus ribavirin.

### Converting microarray datasets into transactional databases

The sequential patterns could be mined directly from a transactional database if the data are discrete. The microarray-involved probe/gene expression values need to be discretized into singleton items within every transaction. Here we show you an example from Table 1 to 3. Table 1 shows the probe/gene expression values of 3 genes *G_1 _*to *G_3 _*over 4 time points *TP_1 _*to *TP_4 _*with a fixed interval (1 day). The experimental design is performed in 3 patients. The first time point of this example is regarded as a baseline for deriving the significant items at each time point. All of the values are then divided by the first time point. The divided values can be presented in a fold change matrix as Table 2. The absolute fold changes exceeding a *fold-change threshold *are further defined as the significant genes. Suppose that the *threshold *is set as 1.5, only the eligible significant genes can be preserved as new items as shown in Table 3. Take patient 1 for instance, up-regulated *G_1_*, down-regulated *G_2 _*and down-regulated *G_3 _*occur at the second time point that will be presented within the same parentheses (transaction). In this example, a set of 3 time-ordered transactions for each patient is called a *sequence*.

**Table 1:** Example of time course microarray dataset

Patient IDs	Genes	TP_1_	TP_2_	TP_3_	TP_4_
1	G_1_	249	656	100	50
	G_2_	333	100	777	989
	G_3_	500	250	157	333

2	G_1_	123	950	135	354
	G_2_	222	987	592	80
	G_3_	300	222	246	735

3	G_1_	500	121	100	50
	G_2_	400	777	520	60
	G_3_	100	300	400	500

TP_n_: gene/probe reading values at time point n.

**Table 2:** Fold changes of gene/probe reading values

Patient IDs	Genes	TP_1/1_	TP_2/1_	TP_3/1_	TP_4/1_
1	G_1_	1.00	2.63	-2.49	-4.98
	G_2_	1.00	-3.33	2.33	2.97
	G_3_	1.00	-2.00	-3.18	-1.50

2	G_1_	1.00	7.72	1.10	2.88
	G_2_	1.00	4.45	2.67	-2.78
	G_3_	1.00	-1.35	-1.22	2.45

3	G_1_	1.00	-4.13	-5.00	-10.00
	G_2_	1.00	1.94	1.30	-6.67
	G_3_	1.00	3.00	4.00	5.00

TP_n/m_: gene/probe reading values of time point n relative to m.

**Table 3:** Converted transactional database

Patient IDs	Sequences
1	<(G_1+_G_2-_G_3-_)_2_(G_1-_G_2+_G_3-_)_3_(G_1-_G_2+_G_3-_)_4_>
2	<(G_1+_G_2+_)_2_(G_2+_)_3_(G_1+_G_2-_G_3+_)_4_>
3	<(G_1-_G_2+_G_3+_)_2_(G_1-_G_3+_)_3_(G_1-_G_2-_G_3+_)_4_>

<>: a sequence; ()_t_: a transaction of time point t; G_+/-_: significantly up- or down-regulated gene item.

However, the content of the converted transactional databases will be affected by different *threshold *settings. In this study, the *threshold *of GSE6377 is set as 1.03 and the *threshold *of GSE11342 is set as 1.5, based on the same criteria used for the original datasets [3,4].

### *CTGR-Span*: cross-timepoint gene regulation sequential pattern

Since the *CTGR-Span *is designed based on a pattern-growth-based manner [9] for mining *CTGR-SPs*, we will present the kernel procedure and meanwhile show the main differences between the traditional pattern-growth-based and our methods using a readily understood example. Finally, we present several extra biologically designed parameters toward more meaningful *CTGR-SPs *in biology.

### Kernel procedure

The main strength of the *CTGR-Span *is to overcome a problem that the transactions have too many items/significant genes. According to our design, it also has several advantages: i) the items within transactions do not need to be sorted in advance, ii) the mining results will not be affected by different sorting types, iii) more meaningful sequential patterns implying gene regulations in biology can be successfully discovered relative to the traditional sequential pattern mining algorithms [7-12], and iv) massive repeated redundant patterns will not be identified. The following examples guide you how to trace the mining processes to explore the patterns from a microarray dataset-converted database. A set *S *of sequences containing 4 patients' transactions is shown in Table 4. Each transaction consists of several significant gene items *G_n+/-_*. In this example, we set a *minimum support *(*minSupp*) as 50%, which means if any one of the items occur in at least 2 different individual sequences (each patient has its own sequence), we call these items as frequent items and further to generate *CTGR-SPs *through a *prefix-projection*-based manner [9] in the following steps:

**Table 4:** Example of transactional database

Patient IDs	Sequences
1	<(G_1+_)_1_(G_2-_G_3+_)_2_(G_3+_)_3_>
2	<(G_1+_G_4-_)_1_(G_3+_)_2_(G_2-_G_3+_)_4_(G_5+_)_5_>
3	<(G_8-_)_1_(G_1+_G_2-_)_2_(G_2-_G_3+_)_3_>
4	<(G_7+_)_1_(G_1+_G_3+_G_6-_)_2_(G_2-_G_3+_)_3_>

<>: a sequence; ()_t_: a transaction of time point t; G_+/-_: significantly up- or down-regulated gene item.

Step 1: Find *length-1 CTGR-SPs*

After scanning the *S*, the frequent items of *length-1 *including <G_1+_>, <G_2-_> and <G_3+_> can be successfully identified since they appear over one half of the sequences. Therefore, these 3 frequent items are regarded as the *lengh-1 CTGR-SPs*.

Step 2: Divide search space

Each item within the set of *length-1 CTGR-SPs *is individually considered as a *prefix *to find its *postfixes *in which they are also frequent in the *S*.

Step 3: Find *postfixes *of *CTGR-SPs*

For each identified *prefix*, the subsets of *CTGR-SPs *can be identified using a depth-first search-based manner in the *prefixes *projected databases.

For readily understanding the above 3 steps, here we extend an example shown in Table II of our previous conference paper [19] as Table 5 and show more descriptions on the comparisons of the traditional sequential pattern-growth-based manner and our proposed *CTGR-Span*. First, for the proposed method, the *prefixes *within *length-1 CTGR-SPs *are shown in the left-most column. Only the subsequences prefixed with the first occurrence of the *prefixes *and started from the next transaction will be presented in the projected databases. As an example, the *prefix *<G_1+_> contained in the sequence <(G_1+_G_4-_)_1_(G_3+_)_2_(G_2-_G_3+_)_4_(G_5+_)_5_> of patient 2 (Table 4), only the subsequence <(G_3+_)_2_(G_2-_G_3+_)_4_(G_5+_)_5_> will be listed in the projected database for mining longer *CTGR-SPs*. According to the same principle, the sequences in *S *containing <G_1+_> are projected to form the <G_1+_>-projected database, which consists of 4 *candidate postfixes*: <(G_2-_G_3+_)_2_(G_3+_)_3_>, <(G_3+_)_2_(G_2-_G_3+_)_4_(G_5+_)_5_>, <(G_2-_G_3+_)_3_> and <(G_2-_G_3+_)_3_>. Then, by scanning <G_1+_>-projected database once, the *length-2 CTGR-SPs *having *prefix *<G_1+_> can be identified including <(G_1+_)(G_2-_)>: 4 (<(G_1+_)(G_2-_)> appears 4 times) and <(G_1+_)(G_3+_)>: 4. The *CTGR-SPs *longer than *length-2 *can be further generated from the current *length-2 CTGR-SPs*. After constructing their respective projected databases, the <(G_1+_)(G_2-_)>-projected database consists of two *candidate postfixes*: <(G_3+_)_3_> and <(G_5+_)_5_>. However, both <(G_3+_)> and <(G_5+_)> appear only once over the sequences involved in the <(G_1+_)(G_2-_)>-projected database that is lower than the *minSupp *(50%). Hence, the further processes for mining the <(G_1+_)(G_2-_)>-projected database will be terminated. On the other hand, recursive mining patterns from the <(G_1+_)(G_3+_)>-projected database, which contains two *candidate postfixes *including <(G_3+_)_3_> and <(G_2-_G_3+_)_4_(G_5+_)_5_>, returns one eligible *postfix *<G_3+_> to form a *length-3 CTGR-SPs *<(G_1+_)(G_3+_)(G_3+_)>. Finally, according to the same criteria, we can find the remaining *CTGR-SPs *prefixed with <G_2-_> or <G_3+_> by constructing their corresponding projected databases.

**Table 5:** Comparison of patterns between a traditional pattern-growth-based approach and CTGR-Span

Prefixes	Traditional projected databases	Projected databases of CTGR-Span	Traditional sequential patterns	CTGR-SPs
G_1+_	<(G_2-_G_3+_)_2_(G_3+_)_3_> <(_G_4-_)_1_(G_3+_)_2_(G_2-_	<(G_2-_G_3+_)_2_(G_3+_)_3_> <(G_3+_)_2_(G_2-_G_3+_)_4_(G_5+_)_5_>	<(G_1+_)(G_2-_)> <(G_1+_)(G_3+_)>	<(G_1+_)(G_2-_)> <(G_1+_)(G_3+_)>
	G_3+_)_4_(G_5+_)_5_>	<(G_2-_G_3+_)_3_>	<(G_1+_)(G_2-_G_3+_)>*	<(G_1+_)(G_3+_)(G_3+_)>
	<(_G_2-_)_2_(G_2-_G_3+_)_3_>	<(G_2-_G_3+_)_3_>	<(G_1+_)(G_3+_)(G_3+_)>	
	<(_G_3+_G_6-_)_2_(G_2-_G_3+_)_3_>			

G_2-_	<(_G_3+_)_2_(G_3+_)_3_>	<(G_3+_)_3_>	<(G_2-_)(G_3+_)>	<(G_2-_)(G_3+_)>
	<(_G_3+_)_4_(G_5+_)_5_>	<(G_5+_)_5_>	<(G_2-_G_3+_)>*	
	<(G_2-_G_3+_)_3_> <(_G_3+_)_3_>	<(G_2-_G_3+_)_3_> <>		

G_3+_	<(G_3+_)_3_>	<(G_3+_)_3_>	<(G_3+_)(G_3+_)>	<(G_3+_)(G_3+_)>
	<(G_2-_G_3+_)_4_(G_5+_)_5_> <>	<(G_2-_G_3+_)_4_(G_5+_)_5_> <>	<(G_3+_)(G_2-_)> <(G_3+_)(G_2-_G_3+_)>*	<(G_3+_)(G_2-_)>
	<(G_6-_)_2_(G_2-_G_3+_)_3_>	<(G_2-_G_3+_)_3_>		

G_+/-_: significantly up- or down-regulated gene item; <>: a sequence; ()_t_: a transaction of time point t; _: indexed prefix; *: redundant patterns derived from traditional pattern-growth-based sequential pattern mining methods.

After mining all of the sequential patterns, apparently, the traditional patterns marked with an asterisk will not be discovered by our proposed method since they contain the simultaneous items at the same time point. For example, in the first row data of Table 5 one <(G_1+_)(G_2-_G_3+_)> out of four traditional sequential patterns contains the simultaneous item G_2- _and G_3+_, which do not imply a gene regulation in a time period but a frequent itemset. Although the pattern could be disassembled into "(G_1+_) → (G_2-_)" and "(G_1+_) → (G_3+_)", they have overlapped with the other explored sequential patterns including the traditional *length-2 *sequential pattern <(G_1+_)(G_2-_)> and <(G_1+_)(G_3+_)>. Therefore, a lot of redundant patterns may be identified by the traditional methods. This thorny problem can be avoided by mining *CTGR-SPs*. Table 5 shows the strength of the *CTGR-Span *and elucidates why *CTGR-Span *is more efficient and useful than the traditional pattern-growth-based methods.

### Biological parameter designs

As stated above, we have introduced the main differences between the traditional and our proposed method. Then we intend to describe how to enrich the patterns with more meaningful in biology. In addition to the inherent parameter *minSupp *for mining traditional patterns, we additionally introduce 3 parameters: *minimum timepoint support *(*minTSupp*), *sliding window size *(*SWS*) and *maximum time constraint *(*maxTC*) to the *CTGR-Span *to mine more meaningful sequential patterns in gene regulation based on some biological characteristics. Since the fundamental definitions of these parameters have been shown in the section II, MATERIALS AND METHODS, of our previous conference paper [19], here we briefly describe their main characteristics and followed by some concrete examples.

**Figure 1:**
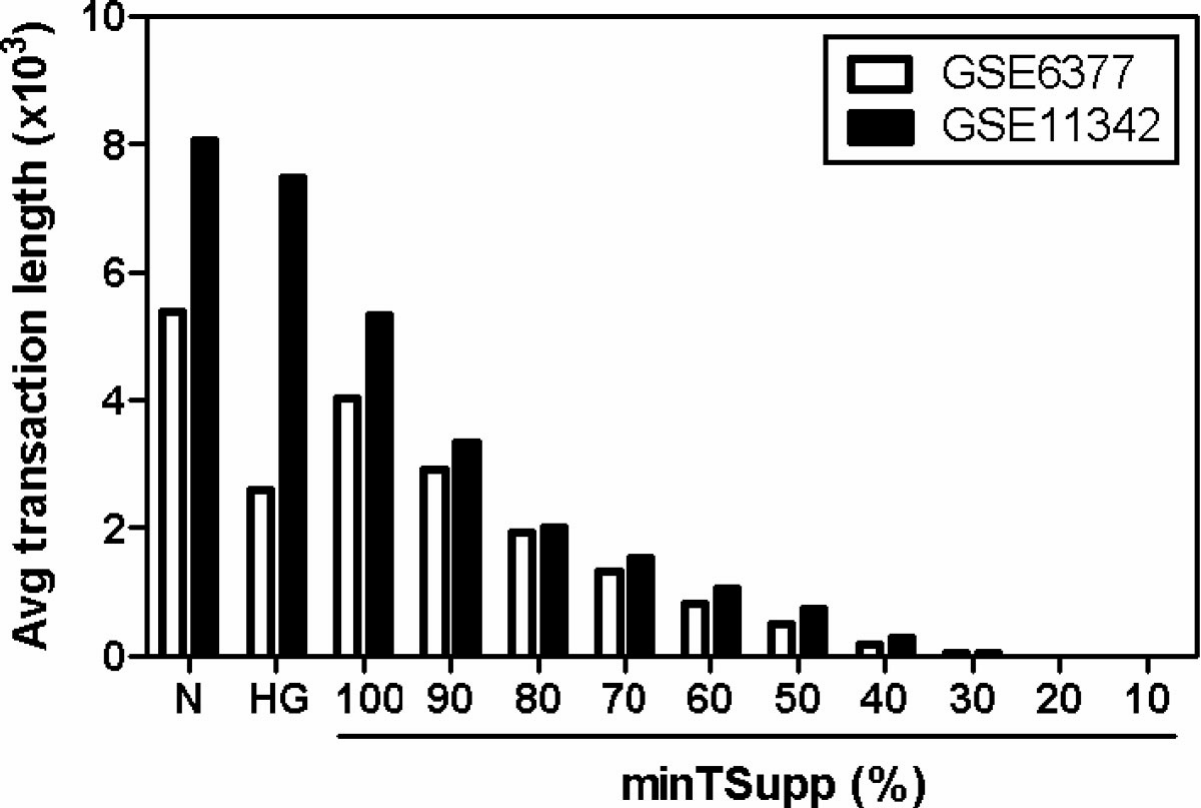
**Average transaction lengths of converted transactional databases**. N: converted transactional databases; HG: filter transactions of the converted transactional databases using a housekeeping gene database.

***minTSupp (minimum timepoint support)***. After converting the input microarray datasets into the transactional datasets, thousands of items are contained in each transaction. The average lengths of the transactions of the two datasets are presented as two bars at the left-most N tick shown in Figure 1. The continuously expressed genes at all-time points may not be susceptible to the cellular responses. They may have a propensity for being housekeeping or maintenance genes [18]. In this regard, some well-studied housekeeping genes (HGs) contained in each transaction will be removed. Based on the similar concept, if the items constitutively appear in most time points, these HG-like items can also be further removed from the transactions using the proposed parameter *minTSupp*. The average lengths of transactions in both input datasets as the functions of varying *minTSupp *are shown in Figure 1.

***SWS (sliding window size)***. Mining sequential patterns implying gene regulations across fixed time points may cause the resulting patterns inadequate because the response times among a set of genes through transcription regulations are not identical. The *sliding window size *(*SWS*) parameter can flexibly allow the patterns containing items to be derived from the same/different time points. Here we show you an example extended from Table 4. Table 6 shows the projected databases of *length-1 CTGR-SPs *when the *SWS *is set as 1. Once the time intervals between the transactions contained in the *length-1*-projected databases and the *prefixes *not exceed 1 (*SWS *= 1), the transactions-involved items and the *prefixes *may actually take place at the same time point. In this case, the gene items involved in a-prime-symbol-marked transactions indicate that they occur with the *prefixes *at the same time point even if all of them originate from different time points.

**Table 6:** Example of *SWS *= 1

Prefixes	Projected databases	CTGR-SPs
G_1+_	<(G_2-_G_3+_)_2'_(G_3+_)_3_>	<(G_1+_G_2-_)>
	<(G_3+_)_2'_(G_2-_	<(G_1+_G_3+_)>
	G_3+_)_4_(G_5+_)_5_>	
	<(G_2-_G_3+_)_3'_>	
	<(G_2-_G_3+_)_3'_>	

G_2-_	<(G_3+_)_3'_>	<(G_2-_G_3+_)>
	<(G_5+_)_5'_> <(G_2-_G_3+_)_3'_>	
	<>	

G_3+_	<(G_3+_)_3'_> <(G_2-_G_3+_)_4_(G_5+_)_5_>	
	<>	<(G_3+_G_3+_)>
	<(G_2-_G_3+_)_3'_>	

G_+/-_: significantly up- or down-regulated gene item; <>: a sequence; ()_t_: a transaction of time point t; _: indexed prefix; *: redundant patterns derived from traditional pattern-growth-based sequential pattern mining methods.

***maxTC (maximum time constraint*)**. Normally, the cells need to react quickly to resist adverse environmental changes, massive short-term gene regulations should be more pronounced within a cellular signaling transduction. In this regard, when setting smaller values of the parameter *maxTC*, a pattern containing two gene items with a big time gap will not be generated. Table 7 shows the *length-1-*projected databases and *CTGR-SPs *from an extended example of Table 4 when *maxTC *is set as 1. The possible *postfixes *for generating *length-2 CTGR-SPs *only will be checked till the transactions marked with a prime symbol.

**Table 7:** Example of *maxTC *= 1

Prefixes	Projected databases	CTGR-SPs
G_1+_	<(G_2-_G_3+_)_2'_(G_3+_)_3_>	<(G_1+_)(G_2-_)>
	<(G_3+_)_2'_(G_2-_G_3+_)_4_(G_5+_)_5_>	<(G_1+_)(G_3+_)>
	<(G_2-_G_3+_)_3'_>	
	<(G_2-_G_3+_)_3'_>	

G_2-_	<(G_3+_)_3'_>	<(G_2-_)(G_3+_)>
	<(G_5+_)_5'_>	
	<(G_2-_G_3+_)_3'_>	
	<>	

G_3+_	<(G_3+_)_3'_>	<(G_3+_)(G_3+_)>
	<(G_2-_G_3+_)_4_(G_5+_)_5_>	
	<>	
	<(G_2-_G_3+_)_3'_>	

G_+/-_: significantly up- or down-regulated gene item; <>: a sequence; ()_t_: a transaction of time point t; _: indexed prefix; *: redundant patterns derived from traditional pattern-growth-based sequential pattern mining methods.

## Results and discussion

In this section, we presented the experimental results of the proposed *CTGR-Span *of two time course gene expression datasets. Because performing the program with different parameter values would yield diverse results, all of the parameters used in this study will be tuned according to the biological backgrounds of the datasets. By introducing the tuned parameter values to the *CTGR-Span*, the resultant *CTGR-SPs *will then be evaluated with previous literature and a GO enrichment analysis to reveal their reliability in biology. Meanwhile, in terms of the performance, the execution efficacy between the traditional and our proposed methods will also be examined in this study.

### Optimal parameter tuning

In addition to the inherent parameter *minSupp *of the traditional methods, we additionally introduced 3 parameters *minTSupp*, *SWS *and *maxTC *to the *CTGR-Span*. However, two questions might arise as to how to set these parameter values for most biologists and whether these parameters are useful for mining gene regulations. In this section, we performed an optimal parameter tuning process to obtain a general rule for setting the parameters without additionally calculating the standard deviations of the time intervals of a dataset in advance [19]. Based on the impact degree of each parameter setting to the numbers of the resulting *CTGR-SPs*, we examined the parameters in an order of *minTSupp *(Table 8 and Supplementary Table 1 to 3 in Additional file 1), *minSupp *(Table 8 and Supplementary Table 1 to 3 in Additional file 1), *maxTC *(Table 9 and 10) and *SWS *(Table 11 and 12). Several characteristics of the mined *CTGR-SPs *of two input datasets are presented in these tables. However, here arises a question as to how to judge which condition (combination of parameter values) is more suitable for further exploration - it is a trade-off that higher parameter values would allow fewer patterns to be mined, but lower parameter values would dramatically increase the number of marginal patterns. Both quantity and quality of the resultant patterns are necessary to be taken into account in this work. In the first dataset (GSE6377), McDunn *et al*. have proven that as the ventilator associated pneumonia (VAP) patients recovered from critical illness complicated by acute infection, the general trajectory (riboleukogram) converged, consistent with an immune attractor [3]. Eighty five genes involved in the inflammatory response were identified with consistent changes in abundance during seven days bracketing the diagnosis of VAP. For the other dataset (GSE11342), Taylor *et al*. identified 85 significantly up/down-regulated genes involved in the immune response from the blood monocytes of hepatitis C patients during the first 10 weeks of treatment with the Peg-interferon alfa-2b plus ribavirin in peripheral [4]. We used a Gene Ontology (GO) enrichment analysis [20] to test if the longest *CTGR-SPs*-involved at least two genes under the conditions are relevant to the corresponding biological manifestations (inflammatory response in GSE6377 and immune response in GSE11342). We focused on the longest *CTGR-SPs *containing at least two gene items because the longer patterns not only contained more significant gene items but also carried more information in a consecutive gene regulation according to the original design of the algorithm. The testing results are presented as -log(p-value) in the tables.

**Table 8:** Characteristics of mined sequential patterns (*minSupp *= variable and *minTSupp *= 100%)

	GSE6377							GSE11342						
		
	100%	95%	90%	85%	80%	75%	70%	100%	95%	90%	85%	80%	75%	70%
# of CTGR-SPs	417	426	4,762	5,090	181,295	181,170	6,948,828	32	224	964	3,077	11,105	6,053	17,412
# of longest CTGR-SPs	81	81	59	59	176,552	176,552	208,297	2	28	203	1,717	4	283	4,713
Maximal length of CTGR-SPs	4	4	6	6	6	6	7	4	4	4	4	5	5	5
# of genes in CTGR-SPs	212	211	1,006	996	2,821	2,826	5,313	25	138	466	1,132	2,011	2,801	4,142
# of genes in longest CTGR-SPs	14	14	11	11	214	214	77	2	3	16	67	3	30	160
# of gene pairs in lonest CTGR-SPs	70	70	58	58	4,077	4,077	1,548	4	21	128	672	6	119	1,119
-Log(p-value)	0.34^†^	0.34^†^	0.00^†^	0.00^†^	0.55^†^	0.55^†^	0.29^† ^	0.00^††^	1.26^††^	0.26^††^	0.91^††^	0.00^††^	1.58^††^	4.11^††^
# of GSP	-	-	-	-	-	-	-	-	-	-	-	-	-	-
# of PrefixSpan	-	-	-	-	-	-	-	-	-	-	-	-	-	-

%: *minSupp *value presented as percentage; †: test longest CTGR-SPs-involved genes in inflammatory response using GO enrichment analysis; ††: test longest CTGR-SPs-involved genes in immune response using GO enrichment analysis; -: no complete patterns.

**Table 9:** Characteristics of mined sequential patterns in GSE6377 (*maxTC *= variable, *minSupp *= 95% and *minTSupp *= 100%)

	2d	3d	4d	5d	6d	7d	8d	9d	≥ 10d
# of CTGR-SPs	157	157	166	166	180	180	298	306	426
# of longest CTGR-SPs	157	157	9	9	17	17	58	58	81
Maximal length of CTGR-SPs	1	1	3	3	4	4	4	4	4
# of genes in CTGR-SPs	157	157	169	169	179	179	201	202	211
# of genes in longest CTGR-SPs	0	0	7	7	10	10	12	12	14
# of gene pairs in lonest CTGR-SPs	0	0	11	11	27	27	50	50	70
-Log(p-value)^†^	-	-	0	0	0	0	0	0	0.34

d: # of days of SWS; †: test longest CTGR-SPs-involved genes in inflammatory response using GO enrichment analysis; -: no p-values.

**Table 10:** Characteristics of mined sequential patterns in GSE11342 (*maxTC *= variable, *minSupp *= 95% and *minTSupp *= 100%)

	28d	31d	34d	37d	40d	43d	46d	49d	52d	55d	58d	61d	64d	≥ 67d
# of CTGR-SPs	112	112	120	126	157	165	160	163	163	161	194	194	220	242
# of longest CTGR-SPs	112	112	8	14	45	2	2	2	2	2	28	28	28	28
Maximal length of CTGR-SPs	1	1	3	3	3	4	4	4	4	4	4	4	4	4
# of genes in CTGR-SPs	112	112	119	123	132	132	132	132	132	132	136	135	136	140
# of genes in longest CTGR-SPs	0	0	4	6	14	2	2	2	2	2	3	3	3	3
# of gene pairs in lonest CTGR-SPs	0	0	7	11	42	4	4	4	4	4	21	21	21	21
-Log(p-value)^††^	-	-	1.02	0.74	0.40	0	0	0	0	0	1.31	1.31	1.31	1.31

d: # of days of SWS; ††: test longest CTGR-SPs-involved genes in immune response using GO enrichment analysis; -: no p-values.

**Table 11:** Characteristics of mined sequential patterns in GSE6377 (*SWS *= variable, *maxTC *= ∞ days, *minSupp *= 95% and *minTSupp *= 100%)

	0d	1d	2d	3d	4d	5d	6d	7d	8d	9d	≥ 10d
# of CTGR-SPs	352	419	203	203	169	169	201	189	279	354	423
# of longest CTGR-SPs	81	81	46	46	3	3	201	189	279	354	423
Maximal length of CTGR-SPs	4	4	3	3	2	2	1	1	1	1	1
# of genes in CTGR-SPs	206	212	178	178	174	174	187	183	197	209	213
# of genes in longest CTGR-SPs	14	14	11	11	2	2	11	9	15	20	21
# of gene pairs in lonest CTGR-SPs	70	70	33	33	5	5	0	0	0	0	0
-Log(p-value)^†^	0.37	0.37	0.44	0.44	0.44	0.44	-	-	-	-	-

d: # of days of SWS; †: test longest CTGR-SPs-involved genes in inflammatory response using GO enrichment analysis; -: no p-values.

**Table 12:** Characteristics of mined sequential patterns in GSE11342 (*SWS *= variable, *maxTC *= ∞ days, *minSupp *= 95% and *minTSupp *= 100%)

	0d	3d	6d	9d	12d	15d	18d	21d	24d	27d	30d	33d	36d	39d	42d	45d	48d	51d	54d	57d	60d	63d	≥ 66d
# of CTGR-SPs	214	211	221	194	154	135	131	127	125	128	125	127	136	157	157	163	163	163	163	187	190	198	217
# of longest CTGR-SPs	28	25	25	82	37	17	17	14	10	13	13	7	10	157	157	163	163	163	163	187	190	198	217
Maximal length of CTGR-SPs	4	4	4	3	3	3	3	3	3	3	2	2	2	1	1	1	1	1	1	1	1	1	1
# of genes in CTGR-SPs	136	134	136	134	127	124	123	121	120	121	119	121	125	132	132	132	132	132	132	136	136	136	136
# of genes in longest CTGR-SPs	3	3	3	15	10	9	9	9	7	8	5	5	4	7	7	7	7	7	7	7	7	7	7
# of gene pairs in lonest CTGR-SPs	21	19	19	59	26	16	16	14	10	12	10	10	12	0	0	0	0	0	0	0	0	0	0
-Log(p-value)^††^	1.26	1.37	1.37	0.70	0.00	0.00	0.00	0.00	0.86	0.86	0.65	0.53	0.40	-	-	-	-	-	-	-	-	-	-

d: # of days of SWS; ††: test longest CTGR-SPs-involved genes in immune response using GO enrichment analysis; -: no p-values.

First of all, if the same significant gene items occur too frequent during a time period, they may be similar to the HGs. Then, the significant patterns should occur as frequently as possible in a group of patients. For these two reasons, we tested both *minTSupp *and *minSupp *from 70% to 100% as shown in Table 8 and Supplementary Table 1 to 3 in Additional file 1. Apparently, the longest *CTGR-SPs *revealed no biologically significant when *minTSupp *was set as 70% or 80% regardless of the values of *minSupp*. Although the *minTSupp *was set as 90%, the common values of *minSupp *suitable for these two input datasets were 85%, 80%, 75% and 70%. Unfortunately, the number of genes involved in the *CTGR-SPs *was too high (over 250 patterns). It might be difficult for most biologists to work with the high number. In spite of these limitations, we could still successfully obtain a suitable common condition for the two datasets when *minTSupp *and *minSupp *were set as 100% and 95%, respectively.

Once the values of *minTSupp *and *minSupp *have been decided, we subsequently tested all possible values of *maxTC *in both two datasets as shown in Table 9 (GSE6377) and Table 10 (GSE11342). The *maxTC *was set from the beginning as largest time interval, 2 days (21-19) in GSE6377 and 28 days (70-42) in GSE11342, to the end as the values which included most transactions bracketing the maximal time interval, 10 days (21-11) in GSE6377 and 67 days (70-3) in GSE11342. For each dataset, the *maxTC *would be increased with the first minimum time interval, 1 day (1-0) in GSE6377 and 3 days (3-0) in GSE11342, to ensure any possible conditions would be tested. Apparently, according to the same criteria mentioned in the above paragraph, there was a suitable common condition for the two datasets when the values of *maxTC *were set as ∞ days.

Finally, we fixed the previous three parameter values and tested the *SWS *as shown in Table 11 (GSE6377) and Table 12 (GSE11342). The values of *SWS *in both datasets were set from the beginning as 0 to the end as the values which included most transactions bracketing the maximal time interval, 10 days in GSE3677 and 66 days in GSE11342. The values of *SWS *were also increased with a fixed interval. Then, we could successfully observe a suitable common condition when the value of *SWS *was set as 3 days. These tables also demonstrate that these suitable common conditions were neither the rule number nor rule length dependent. Incorporating with the domain knowledge of biology to the parameter designs might had a great benefit on discovering the *CTGR-SPs *with potential gene regulations. Therefore, these optimal parameter values could be certainly considered as the default settings to most biologists even if they have no any experiences before.

### High performance of *CTGR-Span*

In this section, we compared the performance of our proposed *CTGR-Span *and the traditional sequential pattern mining algorithms such as the *GSP *and *PrefixSpan *in terms of execution efficiency. For achieving a fair comparison, we performed the *GSP*, *PrefixSpan *and *CTGR-Span *with same parameter settings on both input datasets. The resultant patterns and execution times are presented in Table 8 and Table 13 respectively. However, the traditional algorithms did not allow complete patterns (indicated with "-" in Table 8) to be identified in 2 weeks. Meanwhile, their patterns already have produced tens of millions of patterns. It might be complicated for biologist for find further usage of such massive patters. In contrast, our proposed *CTGR-Span *only needed to take several hours in a worst case that the *minSupp *was set as 70%. (Table 13). These results clearly showed the efficiency of CTGR-Span.

**Table 13:** Execution times (hr) of mined sequential patterns (*minSupp *= variable and *minTSupp *= 100%)

	**GSE6377**							**GSE11342**						
		
	100%	95%	90%	85%	80%	75%	70%	100%	95%	90%	85%	80%	75%	70%
	
GSP	-	-	-	-	-	-	-	-	-	-	-	-	-	-
PrefixSpan	-	-	-	-	-	-	-	-	-	-	-	-	-	-
CTGR-Span	0	0	0.03	0.03	1.65	1.65	220.88	0	0	0	0	0.05	0.23	0.93

%: *minSupp *value presented as percentage; -: over 2 weeks.

### Evaluation with literature

After performing the optimal parameter tuning process, we set the parameter *SWS *= 3 days, *maxTC *= ∞ days, *minSupp *= 95% and *minTSupp *= 100% for the further exploration of *CTGR-SPs *in biology. As stated in the section of optimal parameter tuning, the evaluation criteria for GO enrichment analysis were based on the experimental backgrounds of those two datasets to preliminarily test which condition with longest *CTGR-SPs*-involved genes is much related to the inflammatory response caused by the ventilator-associated pneumonia (GSE6377) and the immune response after drug treatments in hepatitis C patients (GSE11342). In this section, we attempted to further address whether these patterns contain potential genes/regulations which have not been reported in previous literature yet. We scrutinized and evaluated the longest *CTGR-SPs *derived genes from the two input datasets using a manual literature survey. Table 14 and Table 15 show the evaluation results of GSE6377 and GSE11342, respectively. If the patterns contain same items, they will be presented as a single item from left (prefix) to right. For example, in the top-4 data rows of Table 14, there are 4 CAV1_+_-prefixed *CTGR-SPs*: <(CAV1_+_)(GNG7_+_)(EIF2D_+_)>, <(CAV1_+_)(GNG7_+_)(FTSJ2_+_)>, <(CAV1_+_)(GNG7_+_)(NR2E1_-_)> and <(CAV_+_)(GNG7_+_)(TMOD3_-_)>. The CAV1_+ _and GNG7_+ _can be individually grouped and presented as a single item in the table.

**Table 14:** Longest *CTGR-SPs *of GSE6377 (*SWS *= 3 days, *maxTC *= ∞ days, *minSupp *= 95% and *minTSupp *= 100%)

I_1_	I_2_	I_3_	Supports
CAV1+ [21]	GNG7+	EIF2D+ [24]	100% (11/11)
		
		FTSJ2+	100% (11/11)
		
		NR2E1- [22]	100% (11/11)
		
		TMOD3- [25]	100% (11/11)

CCL20- [26]	KIF4A+ [27]	FTSJ2+	100% (11/11)
		
		TMOD3- [25]	100% (11/11)

CSF3R- [28]	GNG7+	CHST7+	100% (11/11)
		
		EIF2D+ [24]	100% (11/11)
		
		FTSJ2+	100% (11/11)
		
		NR2E1- [22]	100% (11/11)
		
		TMOD3- [25]	100% (11/11)
	
	KIF4A+ [27]	FTSJ2+	100% (11/11)
		
		NR2E1- [22]	100% (11/11)
		
		TMOD3- [25]	100% (11/11)

DGKQ+ [29]	GNG7+	FTSJ2+	100% (11/11)

NUDT4+ [30]	CDC25A+ [31]	NR2E1- [22]	100% (11/11)
	
	GNG7+	NR2E1- [22]	100% (11/11)
	
	KIF4A+ [27]	EIF2D+ [24]	100% (11/11)
		
		FTSJ2+	100% (11/11)
		
		NR2E1- [22]	100% (11/11)
		
		SOAT1- [32]	100% (11/11)
	
	TLR6- [33]	CORO1A+ [34]	100% (11/11)
		
		KAT2B- [35]	100% (11/11)
		
		NR2E1- [22]	100% (11/11)
		
		PLAGL1- [22]	100% (11/11)

NUDT4P1+	CDC25A+ [31]	NR2E1- [22]	100% (11/11)
	
	GNG7+	NR2E1- [22]	100% (11/11)
	
	KIF4A+ [27]	EIF2D+ [24]	100% (11/11)
		
		FTSJ2+	100% (11/11)
		
		NR2E1- [22]	100% (11/11)
		
		SOAT1- [32]	100% (11/11)
	
	TLR6- [33]	CORO1A+ [34]	100% (11/11)
		
		KAT2B- [35]	100% (11/11)
		
		NR2E1- [22]	100% (11/11)
		
		PLAGL1- [22]	100% (11/11)

STX4- [36]	CDC25A+ [31]	NR2E1- [22]	100% (11/11)
		
		TMOD3- [25]	100% (11/11)
	
	KIF4A+ [27]	EIF2D+ [24]	100% (11/11)
		
		FTSJ2+	100% (11/11)
		
		NR2E1- [22]	100% (11/11)
		
		TMOD3- [25]	100% (11/11)
	
	TLR6- [33]	CORO1A+ [34]	100% (11/11)
		
		KAT2B- [35]	100% (11/11)
		
		LSM7+ [37]	100% (11/11)
		
		NR2E1- [22]	100% (11/11)
		
		PLAGL1- [22]	100% (11/11)

[]: pneumonia-associated genes reported in previous literature; I_n_: the nth item in a *CTGR-SP*; +: expressed genes; -: repressed genes.

**Table 15:** Longest *CTGR-SPs *of GSE11342 (*SWS *= 3 days, *maxTC *= ∞ days, *minSupp *= 95% and *minTSupp *= 100%)

L_1_	L_2_	L_3_	L_4_	Supports
CXCL10+ [23]	IFIT2+ [38]	ZNF710-	FECH+ [39]	95% (19/20)
			
			BPGM+ [40]	95% (19/20)
			
			SNCA+ [41]	95% (19/20)
			
			SELENBP1+ [42]	95% (19/20)
		
		HBZ+	FECH+ [39]	95% (19/20)
			
			BPGM+ [40]	95% (19/20)
			
			SNCA+ [41]	95% (19/20)
			
			SELENBP1+ [42]	100% (20/20)
			
			TRIM46+	95% (19/20)
		
		SELENBP1+ [42]	HBZ+	95% (19/20)
			
			SELENBP1+ [42]	95% (19/20)
		
		PPP4R4+	SELENBP1+ [42]	95% (19/20)

IFIT2+ [38]	IFIT2+ [38]	ZNF710-	FECH+ [39]	95% (19/20)
			
			BPGM+ [40]	95% (19/20)
			
			SNCA+ [41]	95% (19/20)
			
			SELENBP1+ [42]	95% (19/20)
		
		HBZ+	FECH+ [39]	95% (19/20)
			
			BPGM+ [40]	95% (19/20)
			
			SNCA+ [41]	95% (19/20)
			
			SELENBP1+ [42]	100% (20/20)
			
			TRIM46+	95% (19/20)
		
		SELENBP1+ [42]	HBZ+	95% (19/20)
			
			SELENBP1+ [42]	95% (19/20)
		
		PPP4R4+	SELENBP1+ [42]	95% (19/20)

TNFSF10+ [43]	IFIT2+ [38]	HBZ+	SELENBP1+ [42]	95% (19/20)

[]: hepatitis C-associated genes reported in previous literature; I_n_: the nth item in a *CTGR-SP*; +: expressed genes; -: repressed genes.

After the evaluating process, 78% (54/69 hits) in Table 14 and 73% (29/40 hits) in Table 15 of the patterns-involved genes could be successfully referred to some literature. In other words, the remaining genes might play potential roles during the time course. As stated in the previous example, it has been proven that up-regulated caveolin-1 (CAV1) would regulate NF-kappa B activation and lung inflammatory response to sepsis induced by lipopolysaccharide [21]. The upregulation of nuclear receptor subfamily 2, group E, member 1 (NR2E1) has been revealed by a microarray analysis of mice infected with influenza virus A and Streptococcus pneumonia [22]. A relation/regulation might exist between these two genes since they were strongly related to the pneumonia [21,22]. Coincidentally, in Table 15, upregulated chemokine (C-X-C motif) ligand 10 (CXCL10) has also been reported in the original paper that CXCL10 would be transiently induced early in treatment with Peg-interferon alfa-2b plus ribavirin in peripheral blood monocytes (PBMC) of hepatitis C patients [4]. It could be successfully regarded as plasma indicator for predicting the outcome of antiviral therapy in patients with hepatitis C [23]. Therefore, via this literature evaluation, we postulated that the remaining unreported genes and their relations of the identified patterns in both datasets are highly valuable to be explored in the future.

## Conclusions

In this study, our proposed *CTGR-Span *overcomes the flaws of the traditional sequential pattern mining methods. Although the transactional databases converted from the large-scale time course microarray gene expression datasets have too many items/significant genes within every transaction, the gene regulations over a period of time can still be efficiently identified. The *CTGR-Span *runs dramatically faster than the traditional methods. In addition to the improvement of execution times, we incorporated the characteristics of gene regulation in the parameter designs and further used a GO enrichment analysis to yield the *CTGR-SPs *more meaningful biologically. After evaluating with previous literature, the identified patterns correlate very well with the experimental backgrounds of the two input datasets. Therefore, we postulated that our approach could provide more biological insights into the underlying mechanisms of certain biological or clinical progresses, and it also could be readily applied to other research topics of interest.

## List of abbreviations

CTGR-Span: Cross-Timepoint Gene Regulation Sequential pattern; CTGR-SPs Cross-Timepoint Gene Regulation Sequential Patterns; minTSupp: minimum timepoint support; minSupp: minimum support; SWS: sliding window size; maxTC: maximum time point support; GO: gene ontology.

## Competing interests

The authors declare that they have no competing interests.

## Authors' contributions

CPC, YCL, YLT and VST conceived and designed the entire experiments. CPC carried out the computational studies, performed the statistical analysis and drafted the manuscript. YCL participated in the data interpretations and helped to draft the manuscript. YLT carried out the experiments. VST obtained funding and made critical study supervision. All authors read and approved the final manuscript.

## Supplementary Material

Additional file 1 Characteristics of mined sequential patterns (*minSupp *= 70~100% and *minTSupp *=70%~90%) Click here for file

## Declarations

This research was funded and supported by the National Science Council of Taiwan under contract number "NSC 101-2627-B-006-003" and the National Cheng Kung University Top University Project of Ministry of Education.

This article has been published as part of BMC Bioinformatics Volume 14 Supplement 12, 2013: Selected articles from the IEEE International Conference on Bioinformatics and Biomedicine 2012: Bioinformatics. The full contents of the supplement are available online at http://www.biomedcentral.com/bmcbioinformatics/supplements/14/S12.
