# Supplementary material for: An efficient method for mining cross-timepoint gene regulation sequential patterns from time course gene expression datasets
Source: BMC Bioinformatics. 2013 Sep 24;14(Suppl 12):S3. doi: 10.1186/1471-2105-14-S12-S3 (PMC3848764; doi:10.1186/1471-2105-14-S12-S3)
Supplement: Additional file 1 — Characteristics of mined sequential patterns (minSupp = 70~100% and minTSupp =70%~90%) [file 1471-2105-14-S12-S3-S1.doc]

**Supplementary Tables**

**Table 1.** Characteristics of mined sequential patterns (*minSupp* = variable and *minTSupp* = 90%)

|  | GSE6377 | | | | | | | GSE11342 | | | | | | |  |
| --- | --- | --- | --- | --- | --- | --- | --- | --- | --- | --- | --- | --- | --- | --- | --- |
|  | 100% | 95% | 90% | 85% | 80% | 75% | 70% | 100% | 95% | 90% | 85% | 80% | 75% | 70% | |
| # of CTGR-SPs | 95 | 95 | 1,507 | 1,504 | 8,963 | 9,101 | 29,891 | 3 | 19 | 119 | 284 | 611 | 1,232 | 2,712 | |
| # of longest CTGR-SPs | 30 | 30 | 2 | 2 | 6,411 | 6,411 | 22,501 | 3 | 19 | 119 | 1 | 54 | 1 | 26 | |
| Maximal length of CTGR-SPs | 3 | 3 | 5 | 5 | 5 | 5 | 6 | 1 | 1 | 1 | 3 | 3 | 4 | 4 | |
| # of genes in CTGR-SPs | 84 | 84 | 510 | 510 | 1,602 | 1,607 | 3,280 | 3 | 19 | 119 | 280 | 583 | 1,058 | 1,735 | |
| # of genes in longest CTGR-SPs | 30 | 30 | 6 | 6 | 1,505 | 1,505 | 3,115 | 3 | 19 | 119 | 1 | 35 | 2 | 11 | |
| # of gene pairs in lonest CTGR-SPs | 30 | 30 | 6 | 6 | 1,505 | 1,505 | 3,115 | 0 | 0 | 0 | 2 | 72 | 3 | 40 | |
| -Log(p-value) | 0.47^†^ | 0.47^†^ | 0.47^†^ | 0.11^†^ | 0.11^†^ | 0.11^†^ | 0.31^†^ | - | - | - | 0.87^††^ | 1.68^††^ | 1.68^††^ | 1.68^††^ | |
| %: *minSupp* value; †: test longest CTGR-SPs-involved genes in inflammatory response using GO enrichment analysis; ††: test longest CTGR-SPs-involved genes in immune response using GO enrichment analysis. | | | | | | | | | | | | | | | |

**Table 2.** Characteristics of mined sequential patterns (*minSupp* = variable and *minTSupp* = 80%)

|  | GSE6377 | | | | | | | GSE11342 | | | | | | |  |
| --- | --- | --- | --- | --- | --- | --- | --- | --- | --- | --- | --- | --- | --- | --- | --- |
|  | 100% | 95% | 90% | 85% | 80% | 75% | 70% | 100% | 95% | 90% | 85% | 80% | 75% | 70% | |
| # of CTGR-SPs | 29 | 29 | 287 | 287 | 1,140 | 1,149 | 2,184 | 0 | 3 | 12 | 39 | 107 | 228 | 473 | |
| # of longest CTGR-SPs | 29 | 29 | 111 | 111 | 400 | 400 | 2 | 0 | 3 | 12 | 39 | 107 | 228 | 2 | |
| Maximal length of CTGR-SPs | 1 | 1 | 3 | 3 | 4 | 4 | 6 | 0 | 1 | 1 | 1 | 1 | 1 | 3 | |
| # of genes in CTGR-SPs | 29 | 29 | 221 | 221 | 770 | 774 | 1,717 | 0 | 3 | 12 | 39 | 106 | 223 | 465 | |
| # of genes in longest CTGR-SPs | 0 | 0 | 39 | 39 | 75 | 75 | 4 | 0 | 3 | 12 | 39 | 106 | 223 | 3 | |
| # of gene pairs in lonest CTGR-SPs | 0 | 0 | 127 | 127 | 329 | 329 | 6 | 0 | 0 | 0 | 0 | 0 | 0 | 4 | |
| -Log(p-value) | - | - | 0.00^†^ | 0.00^†^ | 0.00^†^ | 0.00^†^ | 0.00^†^ | - | - | - | - | - | - | 0.00^††^ | |
| %: *minSupp* value; †: test longest CTGR-SPs-involved genes in inflammatory response using GO enrichment analysis; ††: test longest CTGR-SPs-involved genes in immune response using GO enrichment analysis. | | | | | | | | | | | | | | | |

**Table 3.** Characteristics of mined sequential patterns (*minSupp* = variable and *minTSupp* = 70%)

|  | GSE6377 | | | | | | | GSE11342 | | | | | | |
| --- | --- | --- | --- | --- | --- | --- | --- | --- | --- | --- | --- | --- | --- | --- |
|  | 100% | 95% | 90% | 85% | 80% | 75% | 70% | 100% | 95% | 90% | 85% | 80% | 75% | 70% |
| # of CTGR-SPs | 8 | 8 | 73 | 73 | 461 | 461 | 1,313 | 0 | 0 | 2 | 4 | 21 | 49 | 117 |
| # of longest CTGR-SPs | 8 | 8 | 1 | 1 | 163 | 163 | 1 | 0 | 0 | 2 | 4 | 21 | 49 | 117 |
| Maximal length of CTGR-SPs | 1 | 1 | 3 | 3 | 3 | 3 | 5 | 0 | 0 | 1 | 1 | 1 | 1 | 1 |
| # of genes in CTGR-SPs | 0 | 0 | 2 | 2 | 197 | 197 | 4 | 0 | 0 | 0 | 0 | 0 | 0 | 0 |
| # of genes in longest CTGR-SPs | 8 | 8 | 72 | 72 | 375 | 375 | 936 | 0 | 0 | 2 | 4 | 21 | 48 | 114 |
| # of gene pairs in lonest CTGR-SPs | 8 | 8 | 2 | 2 | 197 | 197 | 4 | 0 | 0 | 2 | 4 | 21 | 48 | 114 |
| -Log(p-value) | - | - | 0.00^†^ | 0.00^†^ | 0.00^†^ | 0.00^†^ | 0.00^†^ | - | - | - | - | - | - | - |
| %: *minSupp* value; †: test longest CTGR-SPs-involved genes in inflammatory response using GO enrichment analysis; ††: test longest CTGR-SPs-involved genes in immune response using GO enrichment analysis. | | | | | | | | | | | | | | |
